# Association of Poverty Income Ratio with Metabolic Dysfunction–Associated Steatotic Liver Disease and Liver Fibrosis Among US Population

**DOI:** 10.5152/tjg.2025.25021

**Published:** 2025-06-23

**Authors:** Wenying Guo, Ting Weng, Yufei Song

**Affiliations:** Ningbo Medical Center Lihuili Hospital of Ningbo University, Zhejiang, People’s Republic of China

**Keywords:** Liver fibrosis, MASLD, NHANES, poverty income ratio

## Abstract

**Background:**

**/Aims:** Metabolic dysfunction–associated steatotic liver disease (MASLD) represents a recent update in defining fatty liver disease, emphasizing its strong connection to metabolic factors and reflecting a shift in understanding its causes and progression. The principal aim of this investigation was to scrutinize the conceivable association between the poverty income ratio (PIR) and the incidence of MASLD, specifically focusing on liver fibrosis.

**Materials and Methods:**

In this study, a cross-sectional analysis was carried out utilizing data obtained from the National Health and Nutrition Examination Survey dataset covering the period from 2017 to 2020. To explore the relationship between the PIR and the prevalence of MASLD as well as liver fibrosis, a robust multivariable analytical method was adopted. This approach integrated a wide range of variables, such as sociodemographic characteristics, lifestyle habits, and individual health conditions.

**Results:**

In this study, a comprehensive analysis was conducted using logistic regression models and found a significant decline in the likelihood of MASLD in the highest PIR quartile (Q4) (odds ratio [OR] = 0.634, 95% CI: 0.446-0.903, *P* = .012) as well as liver fibrosis (OR = 0.682, 95% CI: 0.503-0.925, *P* = .014).

**Conclusion:**

The findings obtained from this research strongly demonstrate that higher PIR levels are significantly associated with a reduced prevalence of both MASLD and liver fibrosis, suggesting that higher socioeconomic shighertatus, as reflected by higher PIR, may decrease the risk of these conditions. These findings underscore the need for targeted interventions, such as better nutrition education, lifestyle support, and healthcare access to reduce the MASLD burden in low-income populations.

**Main Points**

- This study marks the initial exploration of the relationship between the poverty income ratio (PIR) and metabolic dysfunction–associated steatotic liver disease, utilizing vibration-controlled transient elastography data derived from the most recent National Health and Nutrition Examination Survey dataset.
- Substantial associations were found between elevated PIR and the presence of liver fibrosis.
- In addition to this, restrictive cubic spline analysis, propensity score matching analysis, and subgroup analysis were conducted to further confirm the results.

## Introduction

Non-alcoholic fatty liver disease (NAFLD) refers to a prevalent liver condition marked by the buildup of lipids within hepatocytes, arising without any association with excessive alcohol intake.^1^ This disorder spans a range, extending from minimal fat accumulation to the more severe phase of non-alcoholic steatohepatitis (NASH). Non-alcoholic fatty liver disease is closely associated with various metabolic disturbances, including obesity, insulin resistance, and type 2 diabetes mellitus (T2DM).^1^ The concept of metabolic dysfunction–associated steatotic liver disease (MASLD) has recently been introduced, offering a clearer framework for categorizing liver disorders by highlighting the metabolic factors that are crucial in the onset of liver steatosis.^2^ Metabolic dysfunction–associated steatotic liver disease refines diagnostic criteria, broadens the spectrum of metabolic conditions linked to liver steatosis, and retains previously established exclusions, thereby facilitating a more inclusive diagnostic approach and enhancing strategies for disease prevention and management.^2^ Epidemiological studies have shown an increasing prevalence of MASLD worldwide, with particularly high rates observed in populations with metabolic risk factors. For instance, a community-based study in Türkiye reported hepatic steatosis in 60.1% of participants,^3^ while a nationwide cohort of T2DM patients found that nearly 70% had MASLD, with a significant proportion at risk of advanced fibrosis.^4^

The PIR serves as a crucial metric employed by the US Census Bureau to measure and assess the economic status of households relative to the federal poverty threshold. Acting as a quantitative measure, this ratio enables an understanding of the economic well-being of individuals and families.^5^ Derived by dividing a household’s income by the applicable poverty threshold corresponding to its size, the PIR provides a standardized indicator of poverty, facilitating meaningful comparisons across diverse demographic cohorts.^6^ Functioning as a dynamic and versatile tool, the PIR substantially contributes to a holistic comprehension of poverty, assuming a pivotal role in the formulation of targeted interventions and policies directed toward enhancing overall societal well-being.^6^

Recognized as the non-invasive benchmark for assessing liver fibrosis, vibration-controlled transient elastography (VCTE) provides enhanced sensitivity and specificity in detecting liver steatosis compared to liver enzyme evaluations, establishing its superiority.^7^ It has been applied to identify liver steatosis and fibrosis in the general population.^7^ Earlier studies have examined the correlation between PIR and hepatic steatosis, utilizing VCTE as a diagnostic technique.^8^ Nonetheless, while PIR’s relationship with liver steatosis has been explored, its correlation with liver fibrosis has been largely overlooked. More importantly, few studies have explored the relationship between PIR and MASLD, a newer and more comprehensive diagnostic category. Unlike NAFLD, which focuses on hepatic fat accumulation, MASLD also provides a more holistic framework for liver disease classification. This study seeks to include participants from the 2017 to 2020 cycle of the National Health and Nutrition Examination Survey (NHANES), which offers the largest VCTE dataset to date. By analyzing this cycle, the study aims to examine the cross-sectional association between PIR, MASLD, and liver fibrosis, providing a more comprehensive understanding of these relationships.

## Materials and Methods

### Study Population

**Figure 1.**
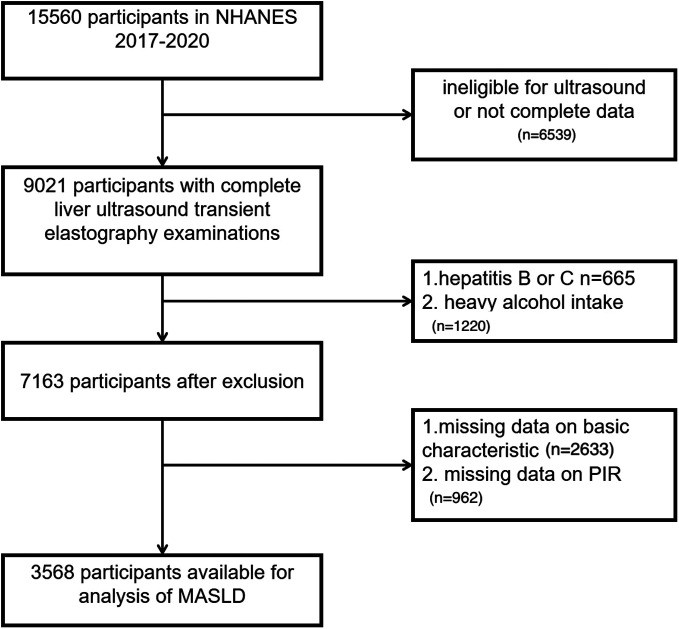
Participant filtering flowchart. Poverty income ratio (PIR).

This study utilized data from the NHANES 2017-2020 cycle. National Health and Nutrition Examination Survey data published online has won the informed consent of participants, so the extra ethical requirements were not needed (details available at https://wwwn.cdc.gov/nchs/nhanes). The study used de-identified NHANES data, which is publicly available and exempt from additional ethics approval. No specific funding was received for this secondary analysis. Initially, 15 560 participants were included in the cohort; however, several exclusions were applied based on the following criteria: (1) Missing liver elastography data (n = 6539), (2) a history of hepatitis B or C diagnosis (n = 638), (3) high alcohol intake, defined as ≥3 drinks per day for men and ≥2 drinks per day for women (n = 1220), (4) absence of demographic or clinical information for the diagnosis of MASLD and logistic regression analysis such as education, marriage, body mass index (BMI), and so on (n = 2633), and (5) incomplete PIR data (n = 962). Following these exclusions, the final analytical cohort included 3568 participants. A comprehensive overview of the participant selection process is illustrated in Figure 1.

### Measurement of Poverty Income Ratio and Vibration-Controlled Transient Elastography

The PIR is determined by dividing the overall yearly income of the family (or individual) by the poverty guidelines corresponding to the survey year. In the household interview, participants were responsible for furnishing the comprehensive income for the entire family in the previous calendar year, denominated in dollars. A family, for the purpose of this definition, refers to a unit consisting of 2 or more people who are connected by blood, marriage, or adoption and reside together under the same roof. Households comprising a single person or unrelated individuals were required to report their annual personal income during the survey. Hepatic steatosis and liver fibrosis were evaluated using VCTE. Consistent with established guidelines, liver steatosis was defined as the value of controlled attenuation parameter (CAP) ≥248 dB/m,^9^ while liver fibrosis was identified by the value of liver stiffness measurement (LSM) value ≥7 kPa.^10^

### Definition of Metabolic Dysfunction–Associated Steatotic Liver Disease

The identification of MASLD predominantly relies on confirming the presence of liver fat accumulation while ruling out excessive alcohol intake or viral hepatitis as potential causes. The inclusion criteria for participants were as follows: (1) A BMI equal to or greater than 25 kg/m2, or a waist measurement (WC) exceeding 94 cm for men and 80 cm for women; (2) Fasting plasma glucose (FPG) levels of at least 5.6 mmol/L, glycated hemoglobin (HbA1c) values of 5.7% or above, a documented history of T2DM, or ongoing management of T2DM; (3) Systolic or diastolic blood pressure readings at or above 130/85 mmHg, or the use of antihypertensive drugs; (4) Triglyceride (TG) concentrations of no less than 1.70 mmol/L, or ongoing therapy for lipid regulation; (5) Levels of high-density lipoprotein cholesterol (HDL-C) below 1.0 mmol/L in males or below 1.3 mmol/L in females, or treatment targeting lipid levels.^2^

### Covariate Assessment

Demographic characteristics were gathered through a structured, self-administered questionnaire, which included details on participants’ age, gender, racial background, educational attainment, marital status, alcohol intake, engagement in physical activities (PA), and current medication usage. Marital status was grouped into 3 categories: “never married,” “married/cohabiting,” and “separated/divorced/widowed.” Educational attainment was classified into 3 levels: “less than high school,” “high school or equivalent,” and “post-high school education.” The level of PA was determined using the formula: PA = metabolic equivalent of task (MET) × weekly frequency × activity duration. A value of zero for PA indicated an absence of physical activity. Participants were categorized based on whether their weekly PA reached the recommended minimum of 600 MET minutes for adults.^11^ Smoking habits were assessed through the analysis of cotinine concentrations: low (serum cotinine <0.015 ng/mL), moderate (0.015-3 ng/mL), and high level (serum cotinine > 3 ng/mL).^12^ Diabetes was identified according to the following parameters: self-reported diabetes diagnosis, the administration of anti-diabetic medications, FPG levels ≥ 7 mmol/L, or HbA1c levels ≥ 6.5%.^13^ Hypertension was defined as systolic or diastolic blood pressure exceeding 130/80 mmHg or the use of blood pressure-lowering treatments.^14^ Alcohol consumption was divided into 2 categories: non-drinkers and moderate drinkers, with moderate drinking defined as 1 drink daily for women as well as 1-2 drinks daily for men.^15^ Missing data for the remaining variables were handled using multiple imputation.

### Statistical Analysis

Continuous variables were presented as means with SDs, while categorical variables were expressed as percentages. Weighted t-tests were used for comparing continuous variables, and chi-square tests were applied to categorical variables. To check for multicollinearity, the variance inflation factor was calculated for each covariate, and all values were below 5, indicating no significant issues with multicollinearity. Three distinct analytical models were developed to examine the complex relationships between covariates and outcomes, with each model incorporating progressively more adjustments. The baseline model remained unchanged, while the second model included adjustments for variables such as PA, BMI, age, ethnicity, sex, education, and marital status. The fully adjusted third model added factors such as smoking status, alcohol consumption, and the presence of diabetes and hypertension. Subgroup analyses were performed to assess potential variations in outcome measures based on gender, age, BMI, diabetes, and hypertension as potential effect modifiers. A restrictive cubic spline (RCS) method was employed to investigate the possible nonlinear relationship between PIR and outcomes associated with MASLD and liver fibrosis. Additionally, propensity score matching and logistic regression analyses were conducted to further explore the link between PIR and MASLD/liver fibrosis. All statistical analyses were carried out using STATA v16.0 (StataCorp LLC, College Station; TX, USA) and R software version 4.1.0 (R Foundation for Statistical Computing; Vienna, Austria).

## Results

### Initial Characteristics of the Study Subjects

A total of 3568 participants were included and categorized into 4 quartile groups based on their PIR levels. Additional information about the baseline characteristics of the participants is provided in Supplementary Table 1. Stratification by PIR revealed significant differences across various factors, including gender, race, educational attainment, marital status, alcohol intake, tobacco use, exercise habits, and the diagnosis of diabetes. Specifically, as PIR increased, participants exhibited higher levels of age, total cholesterol (TC), LDL, and HDL, while showing lower levels of WC, FPG, HbA1c, BMI, CAP, and LSM.

### Association Between Poverty Income Ratio and Metabolic Dysfunction–Associated Steatotic Liver Disease

**Figure 2.**
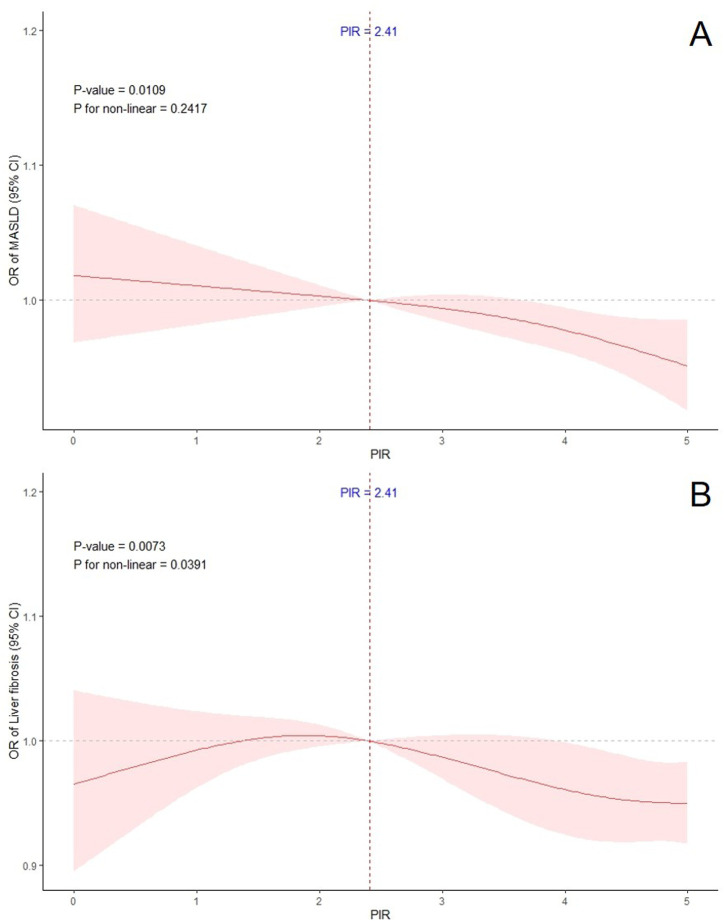
Restrictive cubic spline plots of the association of poverty income ratio with (A) metabolic dysfunction–associated steatotic liver disease, (B) liver fibrosis.

Table 1 displays the outcomes of several logistic regression models, examining the possible distinct relationships between PIR and MASLD. A significant inverse association was observed across all models, particularly in the highest PIR quartile (Q4), with *P*-values of .035 in Model 1, 0.008 in Model 2 (adjusted for demographics), and 0.012 in Model 3 (fully adjusted). The RCS analysis (Figure 2A) demonstrated a linear relationship between PIR and MASLD prevalence, further supporting the robustness of this association.

### Association Between Poverty Income Ratio and Liver Fibrosis

Table 2 presents the findings from various logistic regression models assessing the distinct links between PIR and liver fibrosis. A consistent inverse relationship was found in all models, with statistical significance in the Q4 group (*P* = .028 in Model 1, *P* = .011 in Model 2, and *P* = .014 in Model 3). Unlike the linear trend observed for MASLD, the RCS analysis (Figure 2B) indicated a nonlinear relationship between PIR and liver fibrosis, suggesting potential variations in the effect of PIR at different levels.

### Subgroup Analysis

**Figure 3.**
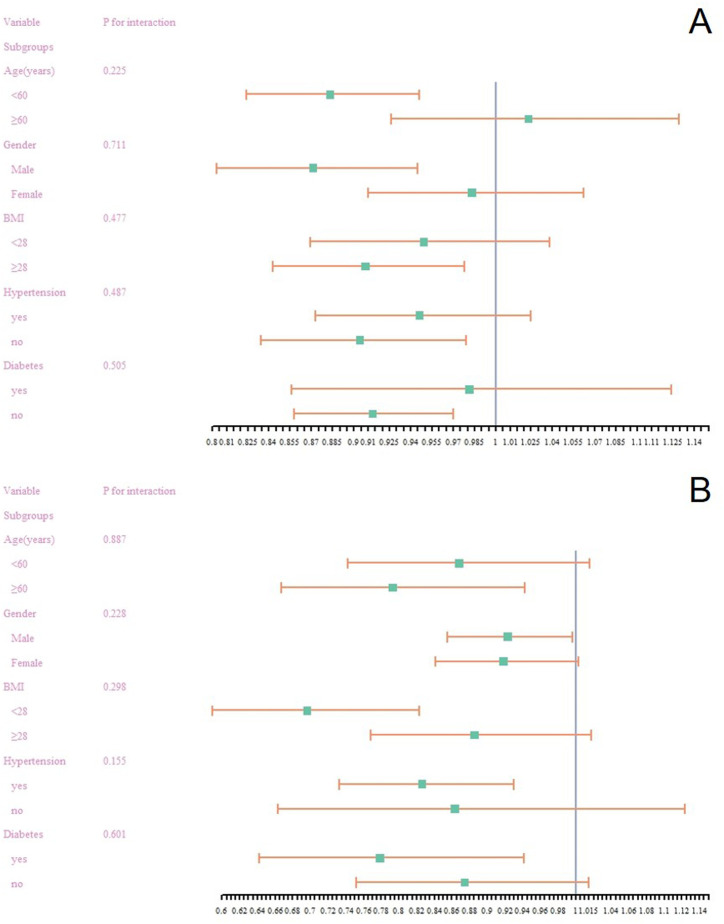
Subgroup analysis of the association between poverty income ratio with (A) metabolic dysfunction–associated steatotic liver disease, (B) liver fibrosis.

Stratified multivariate regression models were employed to investigate the association between PIR and both MASLD as well as liver fibrosis. As depicted in Figure 3, there were no significant relationships identified in any subgroup analysis between PIR and either MASLD or liver fibrosis (all *P* values >.05). To further validate these conditions, propensity score matching was applied followed by logistic regression analysis. The findings (refer to Supplementary Tables 2 and 3) reinforce the consistency and reliability of these results.

## Discussion

Previous investigations have mainly focused on examining the connection between PIR levels and the risk of hepatic steatosis.^8^ Nonetheless, there is a significant lack of epidemiological evidence regarding the possible correlations between PIR levels and both MASLD as well as liver fibrosis. Furthermore, prior studies that employed VCTE to assess the impact of PIR on hepatic steatosis were confined to a single wave of data collection, which led to a relatively limited sample size.^8^ To address these knowledge gaps, an extensive analysis was performed using data from the NHANES surveys conducted between 2017 and 2020, which allowed us to include a broader and more diverse group of participants. These findings revealed a significant and consistent positive association between PIR levels and both MASLD and liver fibrosis throughout the study. Even after accounting for a range of potential confounding variables, such as sociodemographic traits, lifestyle factors, hypertension, and diabetes, these results remained robust. However, subgroup analyses did not reveal any notable differences in grouping factors.

With significant changes observed in dietary habits, urbanization trends, and the rising rates of obesity and T2DM worldwide, particularly in developing countries, the expected trend is an increased global prevalence of NAFLD.^16^ Previous epidemiological investigations have emphasized the intricate correlation between socioeconomic status disparity and metabolic syndrome, with variations observed in this association across regions and countries. These differences are partly associated with genetic predisposition but are also attributed to diverse socioeconomic environments.^17^ In a prior study, considerable regional differences in NAFLD prevalence were identified, with a notable correlation observed with levels of economic and social development. Nations with higher economic standing exhibited a decreased prevalence of NAFLD.^18^ Epidemiological data suggest that demographic cohorts with higher household incomes typically embrace healthier yet more expensive lifestyles from an early stage, whereas households with lower incomes demonstrate increased rates of risky behaviors.^19^ These factors include inadequate access to nutritious food, exposure to harmful environments, and restricted availability of high-quality health services. These factors are closely linked to an increased susceptibility to chronic liver diseases in individuals with lower socioeconomic status.^16^

The economic prosperity of individuals is closely linked to their cognitive behaviors in life, incorporating aspects such as educational achievements, daily exercise habits, and working conditions. Together, these components form what is known as socioeconomic status, representing an individual’s or group’s position within the societal class hierarchy. Fatty liver diseases are more prevalent among individuals with a lower socioeconomic status, as evidenced by limited income, for several reasons.^20^ Initially, individuals who underwent strenuous labor and endured impoverished living conditions during their youth may experience an improvement in living standards with stable incomes post-retirement. However, the inability to adjust lifestyle and dietary patterns exposes them to the risk of NAFLD. The inclination towards excessive consumption of high-fat and high-energy foods, along with heavy alcohol intake, increases their vulnerability to NAFLD.^21^ Additionally, their relatively limited educational attainment impedes their comprehensive understanding of diseases, especially metabolic conditions, which are frequently diagnosed at advanced stages. Their awareness regarding the monitoring and control of blood glucose and blood lipids tends to be relatively deficient.^22^ At the same time, individuals in this demographic display elevated levels of inflammatory markers and insulin resistance, thereby increasing their vulnerability to developing metabolic disorders such as NAFLD. Despite witnessing an income rise, their health awareness remains comparatively suboptimal.^22^

Recent studies have highlighted the increasing prevalence of MASLD among individuals in lower socioeconomic strata. A US study found that food insecurity and low-income households were significant predictors of MASLD, especially when combined with obesity and metabolic syndrome, aligning with these findings.^23^ The relationship between economic disadvantage and liver disease involves several pathways, including diet, stress, healthcare access, and comorbidities. Their study suggests that low-income adolescents are more likely to consume unhealthy, ultra-processed foods, contributing to obesity and insulin resistance—key risk factors for MASLD.^23^ Food insecurity often leads to unhealthy eating patterns, which exacerbate these risks.^24^ Chronic stress, common in low-income households, increases cortisol production, worsening metabolic health and promoting liver disease.^25^ Additionally, low socioeconomic status is associated with higher rates of comorbidities like hypertension and diabetes, which further damage the liver.^26^ Limited healthcare access prevents early diagnosis and adequate management, worsening health outcomes in disadvantaged populations.^27^

In prior subgroup analyses investigating the correlation between PIR and hepatic steatosis, CAP exhibited a notable decline in women compared to men. Earlier epidemiological studies have outlined a significant connection between lower family income during birth and NAFLD in male offspring.^28^ However, the subgroup analysis did not show a substantial association between PIR and MASLD in relation to gender. The discrepancy could be attributed to the focus of the analysis on MASLD as a whole, rather than just isolated CAP measurements. The hypothesis that estrogen could act as a protective factor against NAFLD and that decreased estrogen levels might increase the risk of insulin resistance, cardiovascular diseases, and NAFLD development has been suggested.^29^ In addition to gender, other potential effect modifiers such as age, BMI, diabetes, and hypertension were considered, but no significant differences were found, which is consistent with previous studies.^8^ ^23^ Previous studies found that adolescents with MASLD were more likely to be older than 14 years.^23^ The possible reason for the lack of association with age could be that this study included a broader age range, unlike previous studies that focused specifically on adolescents. Race significantly influences genetic factors, dietary practices, and healthcare access, all of which contribute to the higher incidence of liver disease in certain ethnic groups.^30^ Specifically, previous research indicates that Hispanics are more likely to develop MASLD, with this increased risk attributed to a combination of cultural dietary habits, limited healthcare access, and genetic predisposition.^23^ The lack of significant findings in the subgroup analysis may point to the need for a deeper exploration of why no associations were observed. Potential explanations include health-seeking behaviors, which may vary across subgroups, influencing MASLD detection and management, as well as unmeasured confounders such as genetic factors or environmental exposures. Moreover, the multifactorial nature of MASLD complicates the identification of clear associations, suggesting that PIR alone may not capture the complex interactions influencing liver health. Given the complexity of this relationship, future studies should consider crucial moderators such as age, race/ethnicity, and healthcare access, to provide a more comprehensive understanding of the factors influencing the development of liver diseases. These findings highlight the urgent need for future research to investigate the complex interplay between gender, economic status, and MASLD in subsequent studies.

Based on current knowledge, this investigation represents the first attempt to assess the relationship between PIR levels and both MASLD and liver fibrosis. A comprehensive methodology, including multifactorial regression analysis and subgroup evaluations, was used to examine how PIR levels might influence these conditions. To strengthen the clinical and public health implications of these findings, particularly regarding MASLD screening and prevention among low-income populations, targeted interventions are warranted. Given the higher risk of MASLD and liver fibrosis in individuals with lower PIR, the implementation of cost-effective, non-invasive screening tools such as VCTE should be prioritized in primary care settings to facilitate early detection. Additionally, public health initiatives should focus on improving health literacy by promoting nutritional education, physical activity, and risk factor modification, including smoking cessation and alcohol reduction. Policymakers should also consider subsidizing access to healthier food options, expanding community-based lifestyle intervention programs, and enhancing healthcare accessibility to mitigate the burden of MASLD in socioeconomically disadvantaged groups. Nonetheless, despite the robust design of the study, certain limitations should be addressed. Due to the cross-sectional nature of the NHANES dataset, this study cannot establish a causal relationship between PIR levels and MASLD or liver fibrosis. While a significant inverse association was observed, these findings should be interpreted as correlations rather than direct causation. Unmeasured confounders and reverse causality cannot be ruled out, highlighting the need for longitudinal studies to further clarify this relationship. Even with meticulous control of potential confounding factors, the impact of unaccounted variables on the association between PIR levels and the conditions under investigation cannot be excluded. Although PIR serves as a widely used indicator of socioeconomic status, it may not fully capture other crucial determinants, such as access to healthcare, employment status, or neighborhood socioeconomic conditions, which could influence MASLD risk. These unmeasured factors may contribute to residual confounding, potentially affecting the observed associations. Additionally, while VCTE is a valuable non-invasive tool for assessing liver fibrosis, its diagnostic accuracy can be influenced by BMI, hepatic inflammation, and demographic factors, which may introduce some variability in hepatic steatosis and fibrosis assessment. Moreover, the lack of widely accepted diagnostic standards for liver steatosis and fibrosis through VCTE, along with inconsistencies in the definition of variables such as diabetes, alcohol consumption, and cotinine exposure, introduces some uncertainty in the interpretation of the results.

## Conclusion

In this comprehensive observational study involving a large cohort, a significant correlation between elevated PIR levels and a reduced incidence of MASLD as well as liver fibrosis was observed. Various potential confounding factors were rigorously adjusted for, thereby enhancing the robustness of these associations. These findings highlight the importance of increasing public awareness of the impact of high PIR levels on liver health.

## Supplementary Materials

Supplementary Material

## Figures and Tables

**Table 1.** Logistic Regression Model Between Poverty Income Ratio Level and Metabolic Dysfunction–Associated Steatotic Liver Disease

			Q1	Q2	Q3	Q4
MASLD	Model 1	OR (95% CI)	Reference	0.934 (0.720,1.210)	0.871 (0.669,1.136)	0.808 (0.664,0.985)
		*P*	Reference	.603	.308	.035
	Model 2	OR (95% CI)	Reference	0.841 (0.619,1.142)	0.768 (0.571,1.033)	0.642 (0.462,0.893)
		*P*	Reference	.267	.081	.008
	Model 3	OR (95% CI)	Reference	0.813 (0.588,1.125)	0.767 (0.561,1.048)	0.634 (0.446,0.903)
		*P*	Reference	.212	.096	.012

CI, confidence interval; MASLD, metabolic dysfunction–associated steatotic liver disease; OR, odds ratio.

**Table 2.** Logistic Regression Model Between Poverty Income Ratio Level and Liver Fibrosis

			Q1	Q2	Q3	Q4
Liver fibrosis	Model 1	OR (95% CI)	Reference	1.144 (0.793,1.650)	0.989 (0.694,1.409)	0.662 (0.459,0.956)
		*P*	Reference	.473	.952	.028
	Model 2	OR (95% CI)	Reference	0.990 (0.677,1.449)	0.708 (0.459,1.094)	0.640 (0.453,0.904)
		*P*	Reference	.960	.120	.011
	Model 3	OR (95% CI)	Reference	0.851 (0.612,1.184)	0.751 (0.478,1.179)	0.682 (0.503,0.925)
		*P*	Reference	.339	.213	.014

CI, confidence interval; OR, odds ratio.

**Supplementary Table 1.** Characteristics of Participants Included

Characteristics	Quartile1(<1.24) n=881	Quartile2(1.24-2.40) n=887	Quartile3(2.40-4.44) n=902	Quartile4(≥4.44) n=898	P-value
Age, years	49.31(0.86)	54.15(0.86)	54.08(0.84)	52.88(0.75)	<.001
Gender (n,%)					<.001
Male	390(44.27%)	443(49.94%)	468(51.88%)	508(56.57%)	
Female	491(55.73%)	444(50.06%)	434(48.12%)	390(43.43%)	
Race (n,%)					<.001
Mexican American	117(13.28%)	111(12.51%)	87(9.65%)	49(5.46%)	
Other Hispanic	123(13.96%)	91(10.26%)	72(7.98%)	57(6.35%)	
Non-Hispanic White	222(25.20%)	346(39.01%)	383(42.46%)	401(44.65%)	
Non-Hispanic Black	299(33.94%)	210(23.68%)	213(23.61%)	154(17.15%)	
Other	120(13.62%)	129(14.54%)	147(16.30%)	237(26.39%)	
Education level (n,%)					<.001
Less than high school	304(34.51%)	185(20.86%)	93(10.31%)	19(2.12%)	
High school or equivalent	261(29.63%)	255(28.75%)	187(20.73%)	93(10.36%)	
Above high school	316(35.87%)	447(50.39%)	622(68.96%)	786(87.53%)	
Marital status (n,%)					<.001
Married/cohabitant	399(45.29%)	502(56.60%)	592(65.63%)	680(75.72%)	
Widowed/divorced/separated	249(28.26%)	250(28.18%)	184(20.40%)	125(13.92%)	
Never married	233(26.45%)	135(15.22%)	126(13.97%)	93(10.36%)	
Physical activity (n, %)					<.001
Never	271(30.76%)	279(31.45%)	206(22.84%)	154(17.15%)	
Insufficient	111(12.60%)	94(10.60%)	115(12.75%)	125(13.92%)	
Constant	499(56.64%)	514(57.95%)	581(64.41%)	619(68.93%)	
Drinking status (n,%)					<.001
Non	526(59.70%)	441(49.72%)	390(43.24%)	276(30.73%)	
Low to moderate	355(40.30%)	446(50.28%)	512(56.76%)	622(69.27%)	
Cotinine status (n,%)					<.001
Low	215(24.40%)	315(35.51%)	402(44.57%)	463(51.56%)	
Moderate	402(45.63%)	384(43.29%)	372(41.24%)	340(37.86%)	
High	264(29.97%)	188(21.20%)	128(14.19%)	95(10.58%)	
Diabetes (n,%)					.031
Yes	235(26.67%)	217(24.46%)	205(22.73%)	188(20.94%)	
No	646(73.33%)	670(75.54%)	697(77.27%)	710(79.06%)	
Hypertension (n,%)					.149
Yes	504(57.21%)	536(60.43%)	559(61.97%)	521(58.02%)	
No	377(42.79%)	351(39.57%)	343(38.03%)	377(41.98%)	
BMI,kg/m2 (n,%)					.038
<28	398(45.18%)	408(46.00%)	385(42.68%)	439(48.89%)	
≥28	483(54.82%)	479(54.00%)	517(57.32%)	459(51.11%)	
WC(cm)	101.07(0.86)	100.49(0.84)	101.87(0.78)	98.71(0.75)	.018
TG (mmol/L)	1.18(0.03)	1.20(0.04)	1.24(0.03)	1.18(0.04)	.334
TC (mmol/L)	4.63(0.05)	4.74(0.06)	4.74(0.05)	4.91(0.06)	<.001
LDL (mmol/L)	2.76(0.04)	2.82(0.05)	2.84(0.05)	2.96(0.05)	.031
HDL (mmol/L)	1.33(0.02)	1.37(0.02)	1.34(0.02)	1.41(0.03)	.014
FPG(mmol/L)	6.43(0.11)	6.49(0.11)	6.25(0.08)	6.21(0.09)	.001
Hb1Ac(%)	5.99(0.06)	6.04(0.06)	5.84(0.05)	5.80(0.04)	.002
CAP(dB/m)	267.39(3.33)	267.00(3.47)	266.92(2.86)	265.83(2.92)	.043
LSM(kPa)	5.82(0.18)	5.88(0.24)	5.71(0.15)	5.64(0.22)	<.001
MASLD (n,%)					.177
Yes	505(57.32%)	528(59.53%)	558(61.86%)	517(57.57%)	
No	376(42.68%)	359(40.47%)	344(38.14%)	381(42.43%)	
Liver fibosis (n,%)					.015
Yes	147(16.69%)	150(16.91%)	160(17.74%)	121(13.47%)	
No	734(83.31%)	737(83.09%)	742(82.26%)	777(86.53%)	

**Supplementary Table 2:** . Characteristics of Participants Based on Propensity Score Matching

	Non-MASLD(n=1460)	MASLD(n=1460)	P-value	Non-Liver fibrosis(n=578)	Liver fibrosis(n=578)	P-value
Age (year)	49.96(0.49)	56.77(0.39)	<0.001	57.87(0.96)	56.47(0.95)	.315
Gender(n,%)			<0.001			.405
Male	695(47.60)	801(54.86)		326(56.40)	340(58.82)	
Female	765(52.40)	659(45.14)		252(43.60)	238(41.18)	
Race(n,%)			<0.001			.825
Mexican American	93(6.37)	214(14.66)		65(11.25)	73(12.63)	
Other Hispanic	131(8.97)	160(10.96)		55(9.52)	63(10.90)	
Non-Hispanic White	521(35.68)	618(42.33)		240(41.52)	228(39.45)	
Non-Hispanic Black	436(29.86)	314(21.51)		150(25.95)	151(26.12)	
Other	279(19.11)	154(10.55)		68(11.76)	63(10.90)	
Education level(n,%)			0.004			.907
Less than high school	219(15.00)	276(18.90)		119(20.59)	114(19.72)	
High school or equivalent	319(21.85)	341(23.36)		141(24.39)	146(25.26)	
Above high school	922(63.15)	843(57.74)		318(55.02)	318(55.02)	
Marital status (n, %)			<0.001			.992
Married/cohabitant	822(56.30)	981(67.19)		338(58.48)	340(58.82)	
Widowed/divorced/separated	315(21.58)	344(23.56)		157(27.16)	156(26.99)	
Never married	323(22.12)	135(9.25)		83(14.36)	82(14.19)	
Drinking status (n, %)			0.457			.768
Non	652(44.66)	672(46.03)		269(46.54)	264(45.67)	
Low to moderate	808(55.34)	788(53.97)		309(53.46)	314(54.33)	
Smoking habits (n, %)			<0.001			.239
Never	538(36.85)	632(43.29)		224(38.75)	217 (37.54)	
Former	591(40.48)	601(41.16)		237(41.00)	262(45.33)	
Current	331(22.67)	855(58.56)		117(20.24)	99(17.13)	
Physical activity (n, %)			<0.001			.390
Never	321(21.99)	424(29.04)		178(30.80)	169(29.24)	
Insufficient	173(11.85)	181(12.40)		65(11.25)	80(13.84)	
Constant	966(66.16)	855(58.56)		335(57.96)	329(56.92)	
Diabetes(n, %)			<0.001			.859
Yes	151(10.34)	603(41.30)		260(44.98)	263(45.50)	
No	1309(89.66)	857(58.70)		318(55.02)	315(54.50)	
Hypertension(n, %)			<0.001			.837
Yes	667(45.68)	1138(77.95)		436(75.43)	439(75.95)	
No	793(54.32)	322(22.05)		142(24.57)	139(24.05)	
BMI (kg/m2)			<0.001			.394
<28	1055(72.26)	55(3.77)		121(20.93)	133(23.01)	
≥28	405(27.74)	1405(96.23)		457(79.07)	445(76.99)	

**Supplementary Table 3:** . Logistic Regression Analysis of Between PIR and MASLD, Liver Fibrosis After Propensity Score Matching

			Q1	Q2	Q3	Q4
MASLD	Model1	OR (95%CI)	ref	1.033(0.774,1.380)	1.228(0.920,1.638)	0.768 (0.591, 0.990)
		P trend	ref	0.824	0.163	0.045
	Model2	OR (95%CI)	ref	0.798(0.583,1.094)	0.893(0.644,1.237)	0.668(0.479,0.933)
		P trend	ref	0.161	0.495	0.018
	Model3	OR (95%CI)	ref	0.654(0.399,1.071)	0.705(0.414,1.201)	0.504(0.276,0.919)
		P trend	ref	0.091	0.198	0.025
Liver fibrosis	Model1	OR (95%CI)	ref	1.161(0.734,1.834)	1.113(0.710,1.745)	0.601(0.382,0.945)
		P trend	ref	0.524	0.640	0.028
	Model2	OR (95%CI)	ref	1.120(0.668,1.877)	0.656(0.339,1.269)	0.616(0.407,0.932)
		P trend	ref	0.668	0.210	0.022
	Model3	OR (95%CI)	ref	1.178(0.696,1.995)	0.722(0.361,1.445)	0.608(0.401,0.922)
		P trend	ref	0.541	0.358	0.019

## Data Availability

The data that support the findings of this study are available on request from the corresponding author.
