# Supplementary material for: Association of Poverty Income Ratio with Metabolic Dysfunction–Associated Steatotic Liver Disease and Liver Fibrosis Among US Population
Source: Turk J Gastroenterol. 2025 Jun 23;36(8):488–96. doi: 10.5152/tjg.2025.25021 (PMC12351293; doi:10.5152/tjg.2025.25021)
Supplement: Supplementary Material [file supplementary_material.pdf]

Supplementary Table 1. Characteristics of Participants Included

Characteristics	Quartile1(<1.24) n=881	Quartile2(1.24-2.40) n=887	Quartile3(2.40-4.44) n=902	Quartile4(≥4.44) n=898	P-value
Age, years	49.31(0.86)	54.15(0.86)	54.08(0.84)	52.88(0.75)	<.001
Gender (n,%)					<.001
Male	390(44.27%)	443(49.94%)	468(51.88%)	508(56.57%)	
Female	491(55.73%)	444(50.06%)	434(48.12%)	390(43.43%)	
Race (n,%)					<.001
Mexican American	117(13.28%)	111(12.51%)	87(9.65%)	49(5.46%)	
Other Hispanic	123(13.96%)	91(10.26%)	72(7.98%)	57(6.35%)	
Non-Hispanic White	222(25.20%)	346(39.01%)	383(42.46%)	401(44.65%)	
Non-Hispanic Black	299(33.94%)	210(23.68%)	213(23.61%)	154(17.15%)	
Other	120(13.62%)	129(14.54%)	147(16.30%)	237(26.39%)	
Education level (n,%)					<.001
Less than high school	304(34.51%)	185(20.86%)	93(10.31%)	19(2.12%)	
High school or equivalent	261(29.63%)	255(28.75%)	187(20.73%)	93(10.36%)	
Above high school	316(35.87%)	447(50.39%)	622(68.96%)	786(87.53%)	
Marital status (n,%)					<.001
Married/cohabitant	399(45.29%)	502(56.60%)	592(65.63%)	680(75.72%)	
Widowed/divorced/separated	249(28.26%)	250(28.18%)	184(20.40%)	125(13.92%)	
Never married	233(26.45%)	135(15.22%)	126(13.97%)	93(10.36%)	
Physical activity (n, %)					<.001
Never	271(30.76%)	279(31.45%)	206(22.84%)	154(17.15%)	
Insufficient	111(12.60%)	94(10.60%)	115(12.75%)	125(13.92%)	
Constant	499(56.64%)	514(57.95%)	581(64.41%)	619(68.93%)	
Drinking status (n,%)					<.001
Non	526(59.70%)	441(49.72%)	390(43.24%)	276(30.73%)	
Low to moderate	355(40.30%)	446(50.28%)	512(56.76%)	622(69.27%)	
Cotinine status (n,%)					<.001
Low	215(24.40%)	315(35.51%)	402(44.57%)	463(51.56%)	
Moderate	402(45.63%)	384(43.29%)	372(41.24%)	340(37.86%)	
High	264(29.97%)	188(21.20%)	128(14.19%)	95(10.58%)	
Diabetes (n,%)					.031
Yes	235(26.67%)	217(24.46%)	205(22.73%)	188(20.94%)	
No	646(73.33%)	670(75.54%)	697(77.27%)	710(79.06%)	

(*Continued*)

Supplementary Table 1. Characteristics of Participants Included (*Continued*)

Characteristics	Quartile1(<1.24) n=881	Quartile2(1.24-2.40) n=887	Quartile3(2.40-4.44) n=902	Quartile4(≥4.44) n=898	P-value
Hypertension (n,%)					.149
Yes	504(57.21%)	536(60.43%)	559(61.97%)	521(58.02%)	
No	377(42.79%)	351(39.57%)	343(38.03%)	377(41.98%)	
BMI,kg/m2 (n,%)					.038
<28	398(45.18%)	408(46.00%)	385(42.68%)	439(48.89%)	
≥28	483(54.82%)	479(54.00%)	517(57.32%)	459(51.11%)	
WC(cm)	101.07(0.86)	100.49(0.84)	101.87(0.78)	98.71(0.75)	.018
TG (mmol/L)	1.18(0.03)	1.20(0.04)	1.24(0.03)	1.18(0.04)	.334
TC (mmol/L)	4.63(0.05)	4.74(0.06)	4.74(0.05)	4.91(0.06)	<.001
LDL (mmol/L)	2.76(0.04)	2.82(0.05)	2.84(0.05)	2.96(0.05)	.031
HDL (mmol/L)	1.33(0.02)	1.37(0.02)	1.34(0.02)	1.41(0.03)	.014
FPG(mmol/L)	6.43(0.11)	6.49(0.11)	6.25(0.08)	6.21(0.09)	.001
Hb1Ac(%)	5.99(0.06)	6.04(0.06)	5.84(0.05)	5.80(0.04)	.002
CAP(dB/m)	267.39(3.33)	267.00(3.47)	266.92(2.86)	265.83(2.92)	.043
LSM(kPa)	5.82(0.18)	5.88(0.24)	5.71(0.15)	5.64(0.22)	<.001
MASLD (n,%)					.177
Yes	505(57.32%)	528(59.53%)	558(61.86%)	517(57.57%)	
No	376(42.68%)	359(40.47%)	344(38.14%)	381(42.43%)	
Liver fibosis (n,%)					.015
Yes	147(16.69%)	150(16.91%)	160(17.74%)	121(13.47%)	
No	734(83.31%)	737(83.09%)	742(82.26%)	777(86.53%)	

Supplementary Table 2 . Characteristics of Participants Based on Propensity Score Matching

	Non-MASLD(n=1460)	MASLD(n=1460)	P-value	Non-Liver fibrosis(n=578)	Liver fibrosis(n=578)	P-value
Age (year)	49.96(0.49)	56.77(0.39)	<0.001	57.87(0.96)	56.47(0.95)	.315
Gender(n,%)			<0.001			.405
Male	695(47.60)	801(54.86)		326(56.40)	340(58.82)	
Female	765(52.40)	659(45.14)		252(43.60)	238(41.18)	
Race(n,%)			<0.001			.825
Mexican American	93(6.37)	214(14.66)		65(11.25)	73(12.63)	
Other Hispanic	131(8.97)	160(10.96)		55(9.52)	63(10.90)	
Non-Hispanic White	521(35.68)	618(42.33)		240(41.52)	228(39.45)	
Non-Hispanic Black	436(29.86)	314(21.51)		150(25.95)	151(26.12)	
Other	279(19.11)	154(10.55)		68(11.76)	63(10.90)	
Education level(n,%)			0.004			.907
Less than high school	219(15.00)	276(18.90)		119(20.59)	114(19.72)	
High school or equivalent	319(21.85)	341(23.36)		141(24.39)	146(25.26)	
Above high school	922(63.15)	843(57.74)		318(55.02)	318(55.02)	
Marital status (n, %)			<0.001			.992
Married/cohabitant	822(56.30)	981(67.19)		338(58.48)	340(58.82)	
Widowed/divorced/separated	315(21.58)	344(23.56)		157(27.16)	156(26.99)	
Never married	323(22.12)	135(9.25)		83(14.36)	82(14.19)	
Drinking status (n, %)			0.457			.768
Non	652(44.66)	672(46.03)		269(46.54)	264(45.67)	
Low to moderate	808(55.34)	788(53.97)		309(53.46)	314(54.33)	
Smoking habits (n, %)			<0.001			.239
Never	538(36.85)	632(43.29)		224(38.75)	217 (37.54)	
Former	591(40.48)	601(41.16)		237(41.00)	262(45.33)	
Current	331(22.67)	855(58.56)		117(20.24)	99(17.13)	
Physical activity (n, %)			<0.001			.390
Never	321(21.99)	424(29.04)		178(30.80)	169(29.24)	
Insufficient	173(11.85)	181(12.40)		65(11.25)	80(13.84)	
Constant	966(66.16)	855(58.56)		335(57.96)	329(56.92)	
Diabetes(n, %)			<0.001			.859
Yes	151(10.34)	603(41.30)		260(44.98)	263(45.50)	
No	1309(89.66)	857(58.70)		318(55.02)	315(54.50)	

(Continued)

Supplementary Table 2 . Characteristics of Participants Based on Propensity Score Matching (*Continued*)

	Non-MASLD(n=1460)	MASLD(n=1460)	P-value	Non-Liver fibrosis(n=578)	Liver fibrosis(n=578)	P-value
Hypertension(n, %)			<0.001			.837
Yes	667(45.68)	1138(77.95)		436(75.43)	439(75.95)	
No	793(54.32)	322(22.05)		142(24.57)	139(24.05)	
BMI (kg/m2)			<0.001			.394
<28	1055(72.26)	55(3.77)		121(20.93)	133(23.01)	
≥28	405(27.74)	1405(96.23)		457(79.07)	445(76.99)	

Supplementary Table 3 . Logistic Regression Analysis of Between PIR and MASLD, Liver Fibrosis After Propensity Score Matching

			Q1	Q2	Q3	Q4
MASLD	Model1	OR (95%CI)	ref	1.033(0.774,1.380)	1.228(0.920,1.638)	0.768 (0.591, 0.990)
		P trend	ref	0.824	0.163	0.045
	Model2	OR (95%CI)	ref	0.798(0.583,1.094)	0.893(0.644,1.237)	0.668(0.479,0.933)
		P trend	ref	0.161	0.495	0.018
	Model3	OR (95%CI)	ref	0.654(0.399,1.071)	0.705(0.414,1.201)	0.504(0.276,0.919)
		P trend	ref	0.091	0.198	0.025
Liver fibrosis	Model1	OR (95%CI)	ref	1.161(0.734,1.834)	1.113(0.710,1.745)	0.601(0.382,0.945)
		P trend	ref	0.524	0.640	0.028
	Model2	OR (95%CI)	ref	1.120(0.668,1.877)	0.656(0.339,1.269)	0.616(0.407,0.932)
		P trend	ref	0.668	0.210	0.022
	Model3	OR (95%CI)	ref	1.178(0.696,1.995)	0.722(0.361,1.445)	0.608(0.401,0.922)
		P trend	ref	0.541	0.358	0.019
